# Right internal mammary artery steal syndrome: successful percutaneous coil embolization of a collateral branch—case report

**DOI:** 10.1093/ehjcr/ytag642

**Published:** 2026-09-05

**Authors:** Luca Saccarello, Giovanni Pedrazzini, Marcello Di Valentino, Andrea Demarchi, Andrea Menafoglio

**Affiliations:** Department of Internal Medicine, ORBV, Via A. Gallino 12, 6500 Bellinzona, Switzerland; Department of Pneumology, Universitätsspital Zürich, Rämistrasse 100, Zürich 8091, Switzerland; Cardiocentro Ticino Institute, Ente Ospedaliero Cantonale, Ente Ospedaliero Cantonale, Via Tesserete 48, 6900 Lugano, Switzerland; Faculty of Biomedical Sciences, Università della Svizzera Italiana, Via Buffi 13, 6900 Lugano, Switzerland; Faculty of Biomedical Sciences, Università della Svizzera Italiana, Via Buffi 13, 6900 Lugano, Switzerland; Department of Cardiology, Clinica Luganese Moncucco, Via Soldino 5, 6900 Lugano, Switzerland; Cardiocentro Ticino Institute, Ente Ospedaliero Cantonale, Lugano and Bellinzona, Via Tesserete 48, 6900 Lugano and Via A. Gallino 12, 6500 Bellinzona, Switzerland; Cardiocentro Ticino Institute, Ente Ospedaliero Cantonale, Lugano and Bellinzona, Via Tesserete 48, 6900 Lugano and Via A. Gallino 12, 6500 Bellinzona, Switzerland

**Keywords:** Case report, Coronary artery bypass grafting, Steal syndrome, Right internal mammary artery, Myocardial ischaemia, Coil embolization

## Abstract

**Background:**

Coronary artery bypass grafting (CABG) is a common revascularization strategy, yet ischaemic symptoms may persist despite patent grafts.

**Case summary:**

We describe a 60-year-old man with three-vessel coronary artery disease treated with triple CABG who presented with persistent dyspnoea and inferolateral myocardial hypoperfusion. Repeat coronary angiography demonstrated patent grafts but identified a collateral branch of the right internal mammary artery causing a steal phenomenon. Coil embolization of the collateral vessel led to complete symptom resolution and normalization of myocardial perfusion.

**Discussion:**

This case highlights a rare cause of CABG steal syndrome and emphasizes the need for careful assessment of internal mammary artery branches in patients with ongoing post-CABG ischaemia

Learning points

- Persistent ischaemia after CABG with angiographically patent grafts should prompt evaluation for IMA side-branch steal syndrome.
- Pre-operative selective IMA angiography may help identify significant collateral branches that could cause post-operative flow diversion.
- Percutaneous coil embolization is a safe and effective treatment, with reported success rates exceeding 85%.
- IMA side-branch steal is not limited to LIMA grafts and should also be considered with RIMA conduits.

## Introduction

Bypass graft occlusion is the most frequent complication following coronary artery bypass graft (CABG) surgery. There are however some less common complications. An unusual case is reported of ‘CABG steal syndrome’ by a collateral branch of the right internal mammary artery (RIMA) inducing symptomatic myocardial ischaemia in the inferolateral region. In clinical practice, this entity is seldom described and often leads to diagnostic uncertainty due to its atypical presentation.

## Summary figure

**Figure:**
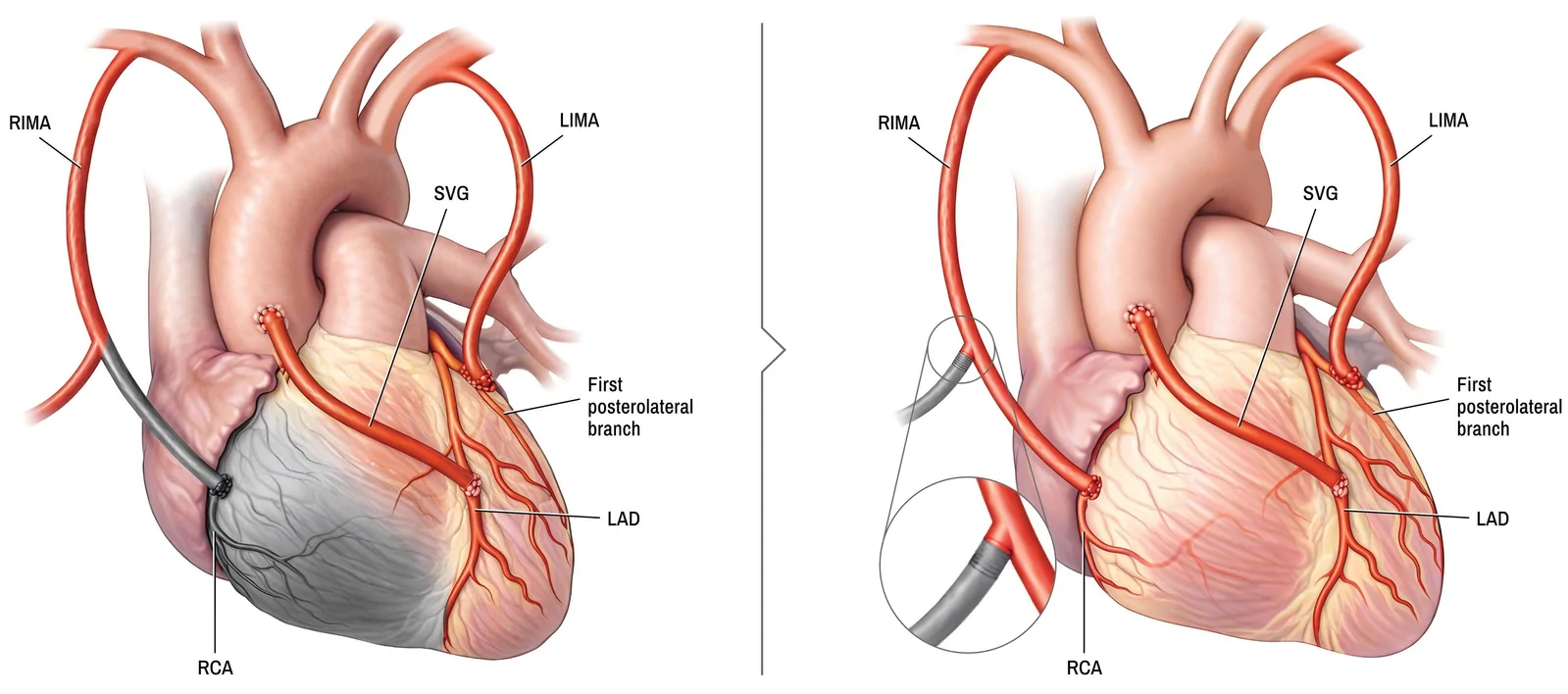
Schematic illustration of CABG anatomy with a side branch of the internal mammary artery causing coronary steal despite patent grafts (left). Selective embolization of the branch restores myocardial perfusion (right).

## Case presentation

A 60-year-old male was referred to our cardiology outpatient clinic after a routine electrocardiogram (ECG) revealed T-wave inversions in leads V3–V5. He presented several cardiovascular risk factors, including arterial hypertension, a positive family history for coronary artery disease, paroxysmal atrial fibrillation, and active heavy tobacco smoking. The patient denied previous known coronary events but reported progressive exertional fatigue over the preceding months.

**Figure 1:**
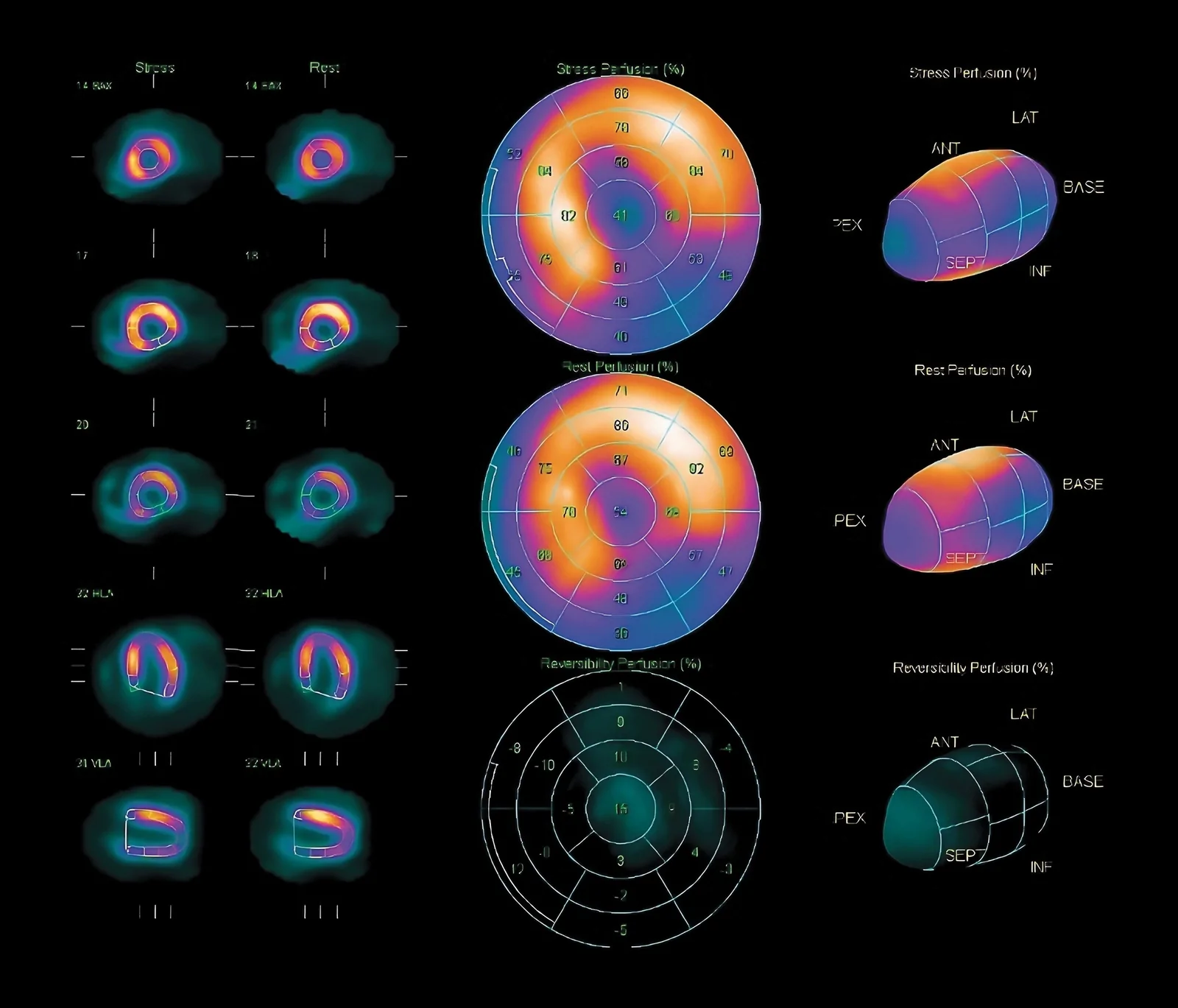
Stress-rest myocardial perfusion scintigraphy showing fixed defects in the inferolateral and apical walls (necrosis) with reversible defects in the anteroseptal and inferolateral perilesional regions (stress-induced ischaemia).

Transthoracic echocardiogram showed hypokinesia of the interventricular septum, the inferior wall, and the inferolateral wall. The exercise stress test was not conclusive for ischaemic ECG changes, though pseudo-normalization of the inverted T-waves developed at peak exertion and persisted during the recovery phase. Myocardial perfusion scintigraphy showed small areas of necrosis in the inferolateral and apical walls, with evidence of stress-induced ischaemia in perilesional areas in the anteroseptal and inferolateral regions (*Figure 1*).

Coronary angiography via right radial access revealed a right-dominant circulation with three-vessel disease involving the left main: 70%–90% ostial left main stenosis, 70%–90% stenosis of the proximal-to-mid left anterior descending artery (LAD) at the bifurcation with the first diagonal branch, 70%–90% stenosis of the first diagonal branch, and 70%–90% long-segment stenosis of the proximal right coronary artery (RCA). The circumflex artery showed no significant disease. Collateral vessels from the RCA territory to the distal LAD were noted. The patient was referred for surgical revascularization and underwent triple CABG: left internal mammary artery (LIMA) to the posterolateral branch, RIMA to the distal RCA, and saphenous vein graft (SVG) with external stenting (VEST) to the mid-LAD.

**Figure 2:**
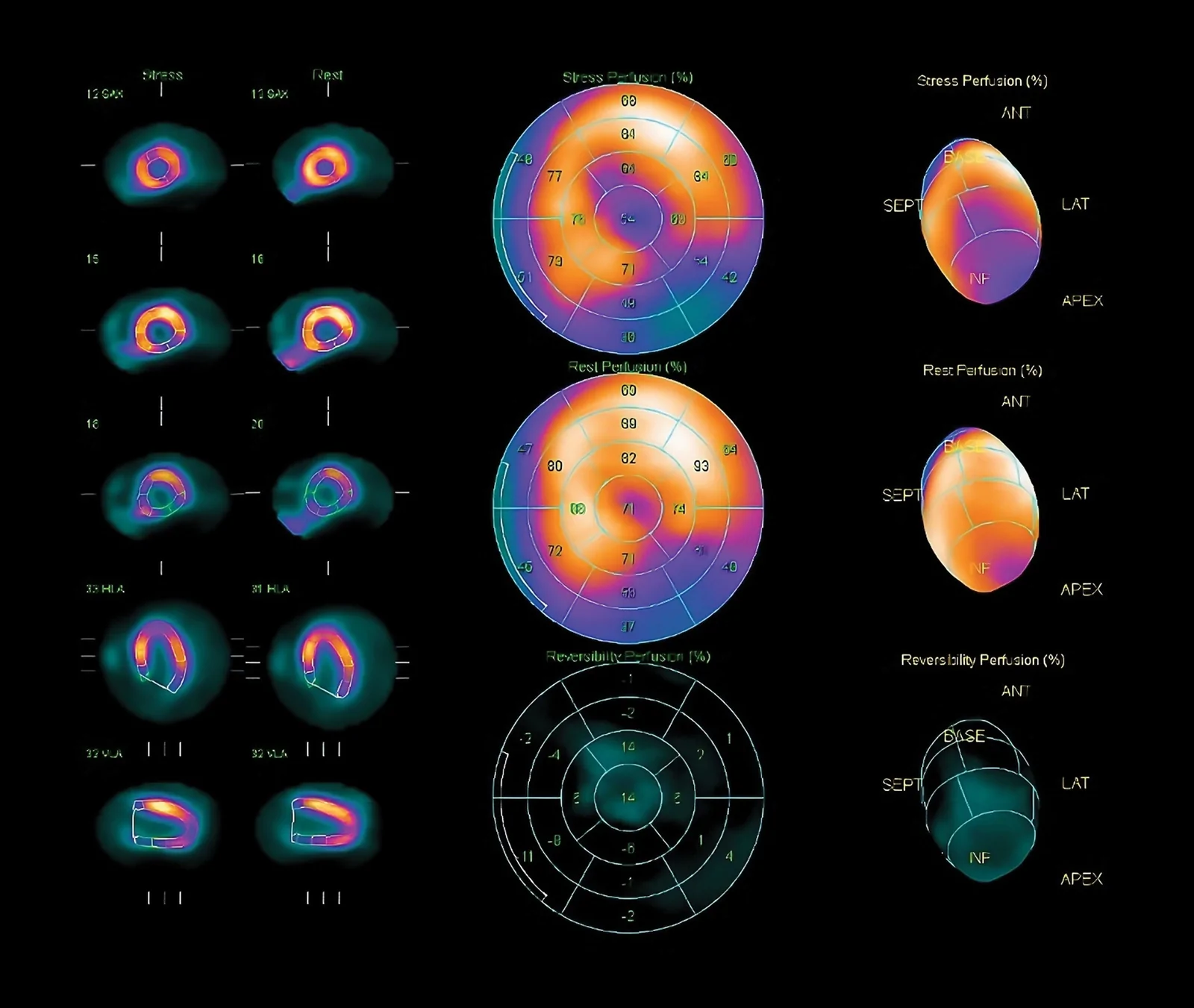
Post-operative stress-rest myocardial perfusion scintigraphy demonstrating persistent hypoperfusion of the inferolateral and apical regions, unchanged from the pre-operative study.

A few weeks post-operatively, the patient still complained of dyspnoea upon mild exertion. Repeat myocardial perfusion scintigraphy showed persistent hypoperfusion of the inferolateral and apical regions, with no improvement compared to the pre-operative study (*Figure 2*). Since the RIMA graft supplied the distal RCA—whose territory encompasses the inferior, inferolateral, and, via collaterals, the apical walls—the persistent ischaemia in these segments raised concern for inadequate graft flow despite anatomical patency.

**Figure 3:**
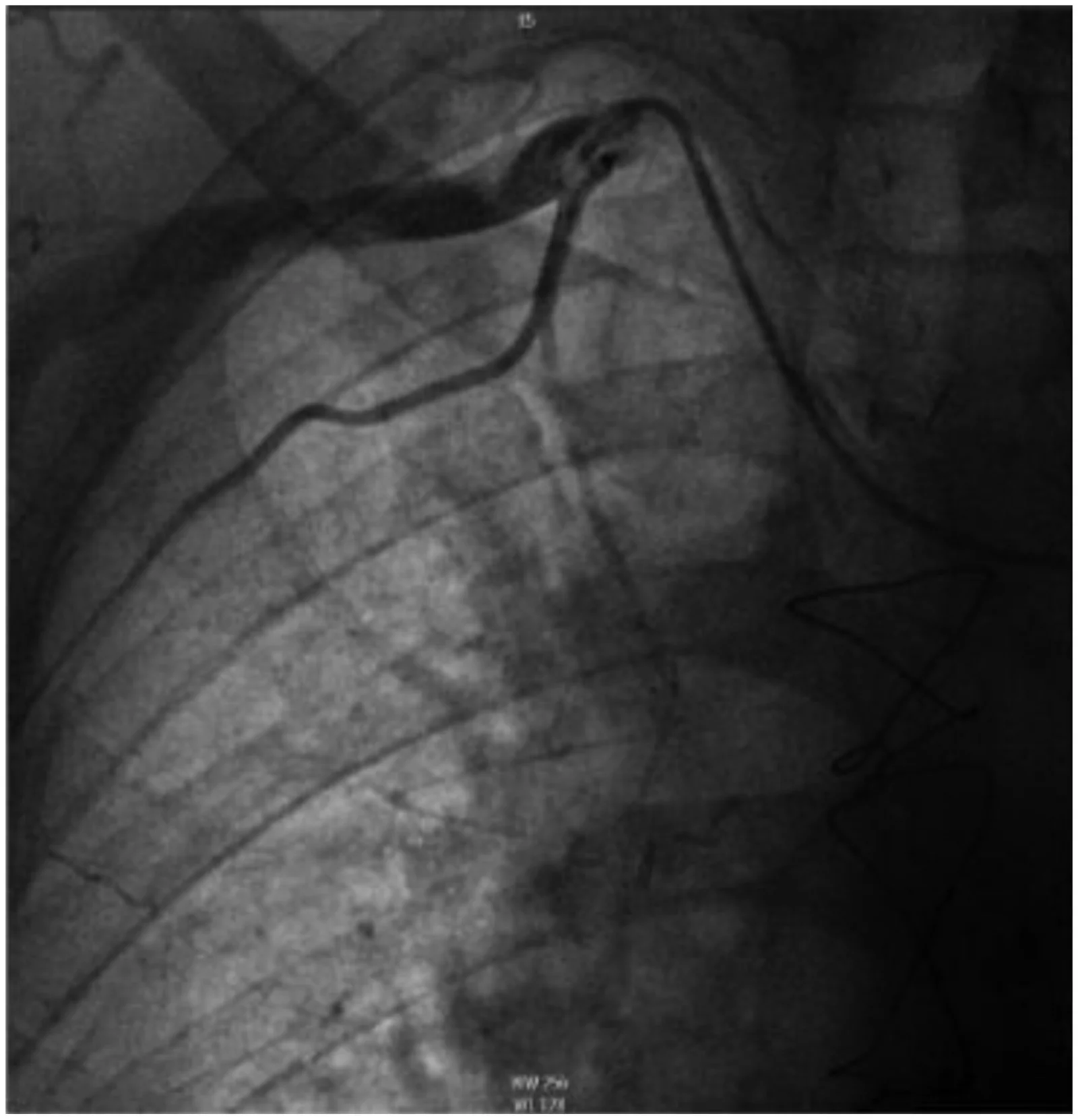
Selective right internal mammary artery graft angiography showing a large proximal side branch with significant flow diversion away from distal right coronary artery.

Repeat coronary angiography confirmed patency of all three bypass grafts with no anastomotic stenoses. However, selective injection of the RIMA graft revealed a large-calibre side branch originating very proximally from the RIMA, diverting an estimated ≥50% of graft flow away from the distal RCA (*Figure 3*). This finding provided a clear anatomical explanation for the persistent inferolateral ischaemia: the steal through the proximal side branch reduced effective perfusion to the RCA territory, which also supplied collaterals to the distal LAD and apical segments. Residual apical ischaemia was additionally attributed to the severely diseased native LAD, which showed chronic total occlusion at the mid-segment with diffuse peripheral disease not amenable to percutaneous intervention.

**Figure 4:**
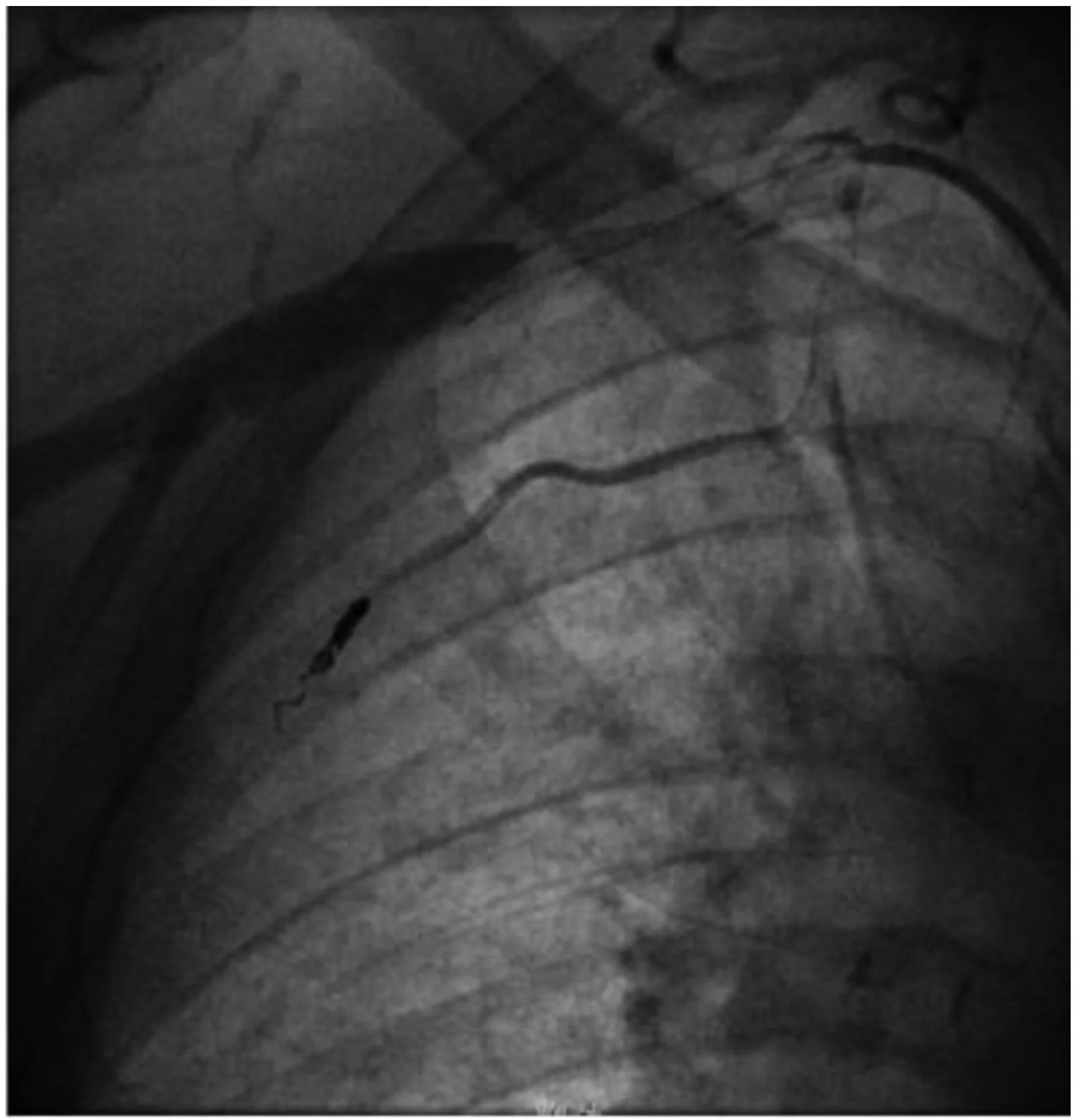
Right internal mammary artery graft angiography immediately after coil deployment. Three coils are visible within the side branch with residual flow prior to complete thrombotic occlusion. Improved antegrade flow to the distal right internal mammary artery is already evident.

One month later, percutaneous embolization of the RIMA side branch was performed via right femoral access. A guide catheter was positioned semi-selectively at the RIMA graft, and a microcatheter was advanced into the side branch. Three detachable coils (3–4 mm diameter) were sequentially deployed with good coil-to-coil interaction. Final angiography showed an intact RIMA graft with preserved flow to the distal RCA; residual flow in the side branch was still present but expected to cease after thrombotic occlusion over the following days. Aspirin was temporarily withheld for 4 days to facilitate thrombosis of the embolized branch (*Figure 4*).

**Figure 5:**
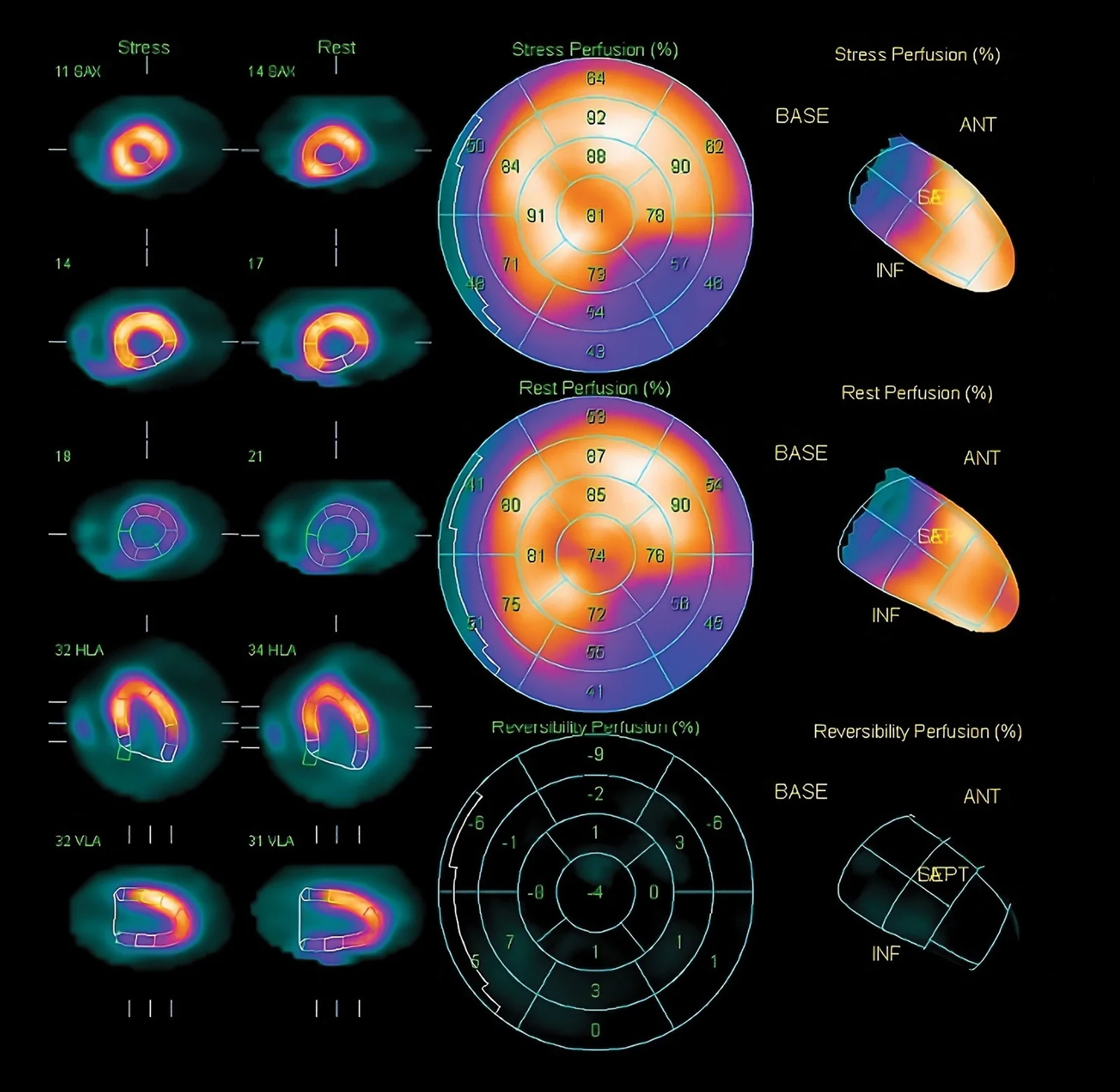
Six-month follow-up myocardial perfusion scintigraphy after coiling, showing resolution of inferolateral hypoperfusion with no evidence of stress-induced ischaemia.

At 6-month follow-up, the patient was completely asymptomatic. Myocardial perfusion imaging showed no stress-induced ischaemia, confirming resolution of the inferolateral hypoperfusion (*Figure 5*). The complete clinical and scintigraphic improvement following selective embolization of the RIMA side branch strongly supported its causal role in the steal phenomenon.

## Discussion

Coronary artery bypass graft is the most commonly performed major cardiac surgical procedure worldwide. According to current European Society of Cardiology guidelines, it remains the preferred revascularization strategy in multivessel coronary artery disease with Synergy Between PCI with TAXUS and Cardiac Surgery (SYNTAX) score ≥ 23, diabetes mellitus, and reduced left ventricular function.^1^ Left internal mammary artery and SVG are the most commonly used conduits; when an additional arterial graft is preferred, both the radial artery and the RIMA may be used. Raja *et al*. suggest that RIMA as a second conduit did not increase operative risk and improved long-term outcomes compared to the radial artery,^2^ while Tector and colleagues suggest preferring the radial artery for right-sided targets because RIMA grafting to the RCA carries a higher occlusion rate, possibly due to kinking by the right lung.^3^

A classical complication of CABG is occlusion of the conduits, which is significantly more frequent in SVGs than in arterial grafts.^4^ A well-recognized cause of recurrent ischaemia with patent grafts is the coronary-subclavian steal syndrome, caused by proximal subclavian artery stenosis resulting in retrograde flow through the internal mammary artery (IMA) graft, typically distinguished by asymmetric arm blood pressures and requiring subclavian revascularization.^4^ A distinct and far less common entity is IMA side-branch steal, in which an unligated side branch of the IMA—intercostal, pericardiophrenic, or musculophrenic—creates a low-resistance pathway that diverts blood flow away from the coronary target.^5,6^

Whether this steal is clinically significant remains debated. Guzon *et al*. found no significant changes in LIMA flow after balloon occlusion of thoracic side branches.^7^ Kern argued that phasic flow patterns make true steal physiologically unlikely.^8^ Conversely, Derimay *et al*. provided compelling evidence using fractional flow reserve, showing improvement from 0.76 to 0.85 after coil embolization of an intercostal branch.^9^ These conflicting findings suggest that IMA steal occurs in specific anatomic and haemodynamic conditions, particularly when side branches are large and distal coronary bed resistance is high.^6^

Since the first descriptions in the 1990s, IMA side-branch steal has been reported in a growing number of publications. The most comprehensive review by Mangels *et al*. identified at least 44 patients across 31 publications who underwent therapeutic closure of LIMA side branches, with 87.5% achieving freedom from angina.^6^ Key cases include the following: Chavan *et al*. treated five patients with coil embolization with angina resolution in three^10^; Hartz and Heuser documented ischaemia resolution after embolization of a large LIMA first intercostal branch^11^; Pagni *et al*. reported recanalization after coil embolization requiring video-assisted thoracoscopic surgery (VATS) ligation^12^; Weinberg *et al*. first reported the use of the Amplatzer Vascular Plug^13^; and Picci *et al*. reported successful coil embolization of a large LIMA side branch.^14^ Abdelfattah *et al*., in a single-centre 12-year experience, reported a 100% procedural success rate in seven patients undergoing transcatheter coil embolization.^15^ Percutaneous coil embolization remains the most commonly reported treatment, with VATS ligation as a surgical alternative.^6,12^

Several alternative causes of persistent post-CABG ischaemia with patent grafts were systematically considered. Progression of native coronary disease was excluded by repeat angiography. Coronary-subclavian steal syndrome was ruled out by the absence of asymmetric arm blood pressures. Competitive flow was considered unlikely given the severity of the native RCA stenosis. Microvascular dysfunction was deemed less probable given the complete resolution of symptoms after embolization of the RIMA side branch alone.^1^

To our knowledge, all previously published cases of IMA side-branch steal involved the LIMA, as confirmed by the comprehensive review by Mangels *et al*. which identified 44 patients across 31 publications, all involving LIMA side branches.^6^ The present case represents the first report of this entity involving a RIMA side branch, extending the clinical spectrum of IMA side-branch steal to bilateral mammary artery grafts.

The successful treatment with coil embolization and complete symptom resolution at 6 months is consistent with outcomes reported in LIMA steal cases. This case reinforces the importance of considering IMA side-branch steal in the differential diagnosis of recurrent ischaemia after CABG when grafts are patent.

Unfortunately, this complication is difficult to predict, and complete harvesting of the RIMA or LIMA is not recommended given its rarity.^5,6^ However, during pre-CABG coronary angiography, selective imaging of the RIMA and LIMA should not only confirm patency of these arteries, but it may also be worthwhile to carefully look for significant collateral vessels. Identifying such branches beforehand may occasionally help prevent post-operative diagnostic ambiguity and guide optimal conduit selection.

## Lead author biography


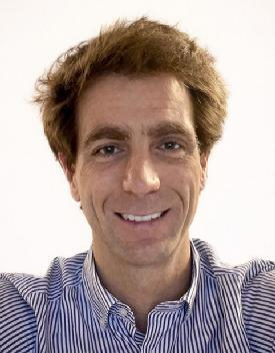


Born in Genoa on 8 August 1991, I studied Medicine at the University of Genoa, graduating in 2017 with honours (110/110 cum laude). I completed a clinical internship at Hôpital l’Archet in Nice, France. I began my postgraduate training in Internal Medicine in Switzerland, working across the hospitals of Locarno, Mendrisio, and Bellinzona, where I also completed a rotation in Cardiology. After completing a 1-year rotation in Pulmonology, I obtained board certification in Internal Medicine. I am currently pursuing a second specialization in Pulmonology at the University Hospital Zurich, focusing on clinical practice and research in respiratory medicine and physiology.

## Data Availability

The data underlying this article will be shared on reasonable request to the corresponding author.

## References

[1] Neumann FJ, Sousa-Uva M, Ahlsson A, Alfonso F, Banning AP, Benedetto U, et al 2025 ESC guidelines for the management of myocardial revascularization. Eur Heart J 2025;46:3952–4041.40878297

[2] Raja SG, Benedetto U, Amrani M. Right internal mammary artery versus radial artery as second arterial conduit in coronary artery bypass grafting: a case-control study of 1526 patients. Int J Surg 2015;16:183–189. 25153938 10.1016/j.ijsu.2014.08.342

[3] Tector AJ . Fifteen years’ experience with the internal mammary artery graft. Ann Thorac Surg 1986;42:S22–S27. 2878648 10.1016/s0003-4975(10)64636-x

[4] Cua B, Mamdani N, Halpin D, Jhamnani S, Jayasuriya S, Mena-Hurtado C. Review of coronary subclavian steal syndrome. J Cardiol 2017;70:432–437. 28416323 10.1016/j.jjcc.2017.02.012

[5] Moreno N, Castro da Silva A, Pereira A, Silva J, Almeida P, Andrade A, et al Ischemia induced by coronary steal through a patent mammary artery side branch: a role for embolization. Catheter Cardiovasc Interv 2004;61:558–561. 10.1016/j.repc.2012.09.011 23809629

[6] Mangels D, Penny W, Reeves R. Left internal mammary artery side branch intervention in the management of coronary steal syndrome following coronary artery bypass grafting. Catheter Cardiovasc Interv 2021;97:97–104. 31782888 10.1002/ccd.28630

[7] Guzon OJ, Klatte K, Moyer A, Khoukaz S, Kern MJ. Fallacy of thoracic side-branch steal from the internal mammary artery: analysis of left internal mammary artery coronary flow during thoracic side-branch occlusion with pharmacologic and exercise-induced hyperemia. Catheter Cardiovasc Interv 2004;61:20–28. 14696154 10.1002/ccd.10722

[8] Kern MJ . Is stealing still a crime? Comment on left internal mammary artery side branch intervention in the management of coronary steal syndrome following coronary artery bypass grafting. Catheter Cardiovasc Interv 2021;97:105–107. 33460263 10.1002/ccd.29446

[9] Derimay F, Hayek A, Rioufol G. Coronary steal after left internal thoracic artery grafting: demonstration and management with fractional flow reserve. JACC Cardiovasc Interv 2021;14:2519–2520. 34756541 10.1016/j.jcin.2021.08.074

[10] Chavan A, Mügge A, Hohmann C, Raab BW, Jantsch H, Galanski M. Recurrent angina pectoris in patients with internal mammary artery to coronary artery bypass: treatment with coil embolization of unligated side branches. Radiology 1996;200:433–436. 8685338 10.1148/radiology.200.2.8685338

[11] Hartz RS, Heuser RR. Embolization of IMA side branch for post-CABG ischemia. Ann Thorac Surg 1997;63:1765–1766. 9205181 10.1016/s0003-4975(97)00434-7

[12] Pagni S, Bousamra M, Shirley MW, Spence PA. Successful VATS ligation of a large anomalous branch producing IMA steal syndrome after MIDCAB. Ann Thorac Surg 2001;71:1681–1682. 11383825 10.1016/s0003-4975(00)02300-6

[13] Weinberg N, French WJ, Shavelle DM. Use of the amplatzer vascular plug for treatment of coronary steal. Int J Cardiol 2008;124:109–111. 17379329 10.1016/j.ijcard.2006.11.203

[14] Picci A, Certo G, Ceresa F, Patanè F. A case of LIMA side branch coronary steal syndrome: a role for embolization. Catheter Cardiovasc Interv 2024;104:1217–1219. 39413267 10.1002/ccd.31270

[15] Abdelfattah OM, Saad AM, Kassis N, Shekhar S, Isogai T, Gad MM, et al Utilization and outcomes of transcatheter coil embolization for various coronary artery lesions: single-center 12-year experience. Catheter Cardiovasc Interv 2021;98:1317–1331. 33205571 10.1002/ccd.29381

